# Superimposition of checkerboard distribution of ephelides and neurofibromas in a patient with segmental neurofibromatosis

**DOI:** 10.1016/j.jdcr.2022.05.025

**Published:** 2022-05-30

**Authors:** Hisato Iriki, Noriko Umegaki-Arao, Risa Kakuta, Harumi Fujita, Satomi Aoki, Masayuki Amagai, Takashi Sasaki, Yasuo Hamamoto, Robert Nakayama, Akiharu Kubo

**Affiliations:** a Department of Dermatology, Keio University School of Medicine, Tokyo, Japan; b Department of Dermatology, Tokyo Women's Medical University Adachi Medical Center, Tokyo, Japan; c KOSÉ Endowed Program for Skin Care and Allergy Prevention, Keio University School of Medicine, Tokyo, Japan; d Center for Supercentenarian Medical Research, Keio University School of Medicine, Tokyo, Japan; e Keio Cancer Center, Keio University School of Medicine, Tokyo, Japan; f Department of Orthopaedic Surgery, Keio University School of Medicine, Tokyo, Japan; g Division of Dermatology, Department of Internal Related, Kobe University Graduate School of Medicine, Kobe, Japan

**Keywords:** checkerboard pattern, mosaic mutation, prenatal somatic mutation, segmental neurofibromatosis, GIST, gastrointestinal stromal tumor, NF1, neurofibromatosis type I, PNR, peripheral nerve-related, SNF, segmental neurofibromatosis

## Introduction

Segmental neurofibromatosis (SNF) is a mosaic form of neurofibromatosis type I (NF1) caused by a prenatal somatic mutation in *NF1.*^1^ Various tumors can develop in patients with SNF and systemic neurofibromatosis, including neurofibromas, malignant peripheral nerve sheath tumors, and gastric adenocarcinomas.^2^^,^^3^ SNF tumors originate from cells that bear a prenatal *NF1* mutation. Mosaic skin disorders exhibit several characteristic patterns of skin lesions, including Blaschko-linear, checkerboard, phylloid, and patchy patterns, depending on the cell type that bears the causative mutation.^4^ Most patients with SNF are known to exhibit a segmental, checkerboard pattern, a distribution of café-au-lait spots, ephelides, and/or neurofibromas.^1^ The numbers and distributions of segmental lesions differ among patients. Ephelides/café-au-lait spots and neurofibromas are considered to originate from *NF1*-mutated neural crest cells that differentiate into melanocytes and peripheral nerve-related (PNR) cells, respectively.^5^^,^^6^ Here, we encountered an SNF patient who exhibited segmentally distributed ephelides and neurofibromas with independent, but partially superimposed, checkerboard patterns.

## Case report

**Fig 1:**
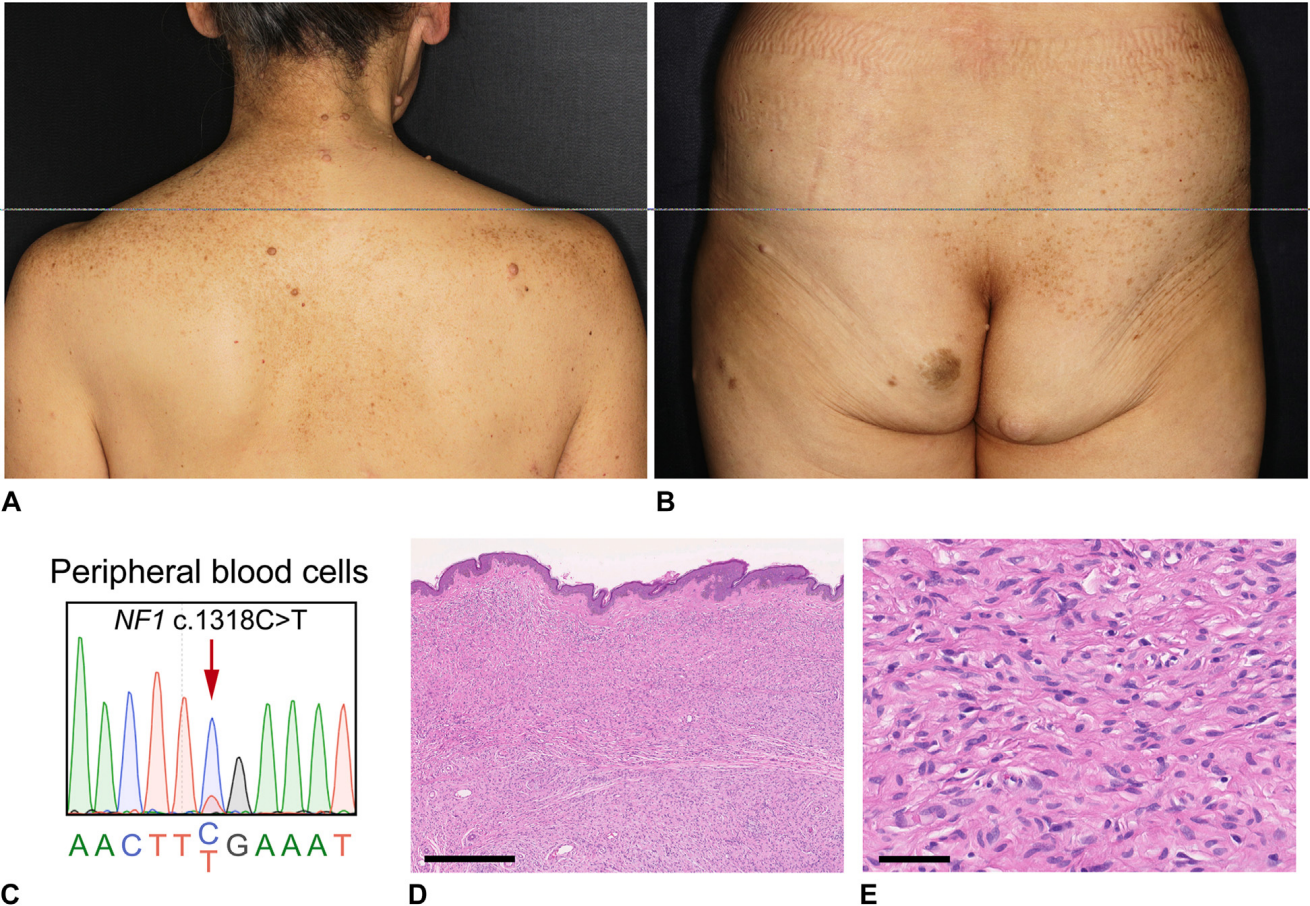
Clinical phenotype and pathology. **A, B,** Independent checkerboard patterns of ephelides and neurofibromas on the upper back (**A**) and buttocks (**B**). **C,** A Sanger sequencing chromatogram showing the *NF1* mosaic mutation (*red arrow*) c.1318C>T (p. Arg 440∗) in peripheral blood cells. **D, E,** Hematoxylin-eosin staining of the tumors. Low-power (**D**) and high-power (**E**) views revealed small spindle cells and wavy tumor fibrils (**E**). Scale bars, 500 μm (**D**) and 50 μm (**E**).

A 61-year-old woman presented to the dermatology clinic with multiple café-au-lait spots that had appeared during childhood, segmental ephelides, and multiple adult-onset soft tumors. She had developed an malignant peripheral nerve sheath tumor in the left part of her neck and multiple gastrointestinal stromal tumors (GISTs) of the stomach at the age of 60 years. She had no family history of neurofibromatosis. The ephelides were distributed from the left part of her neck to her left shoulder and on the right upper part of her back and right hip, whereas the soft tumors were distributed from the right part of her neck to the right shoulder and on both sides of the upper part of her back and hips (Fig 1, *A* and *B*). A skin biopsy of a soft tumor on her back revealed a well-circumscribed proliferation of small spindle cells, indicating a neurofibroma. After obtaining written informed consent approved by the Institutional Review Board of the Keio University School of Medicine, we performed Sanger sequencing of genomic DNA from peripheral blood leukocytes and identified a somatic mosaicism (c.1318C>T [p.R440∗] mutation) in *NF1* (NM_000267.3) (Fig 1, *C*). A comparison of the Cytosine and Thymine peaks in the Sanger sequencing chromatogram suggested that the frequency of the mutant allele was ∼20%. We diagnosed SNF.

**Fig 2:**
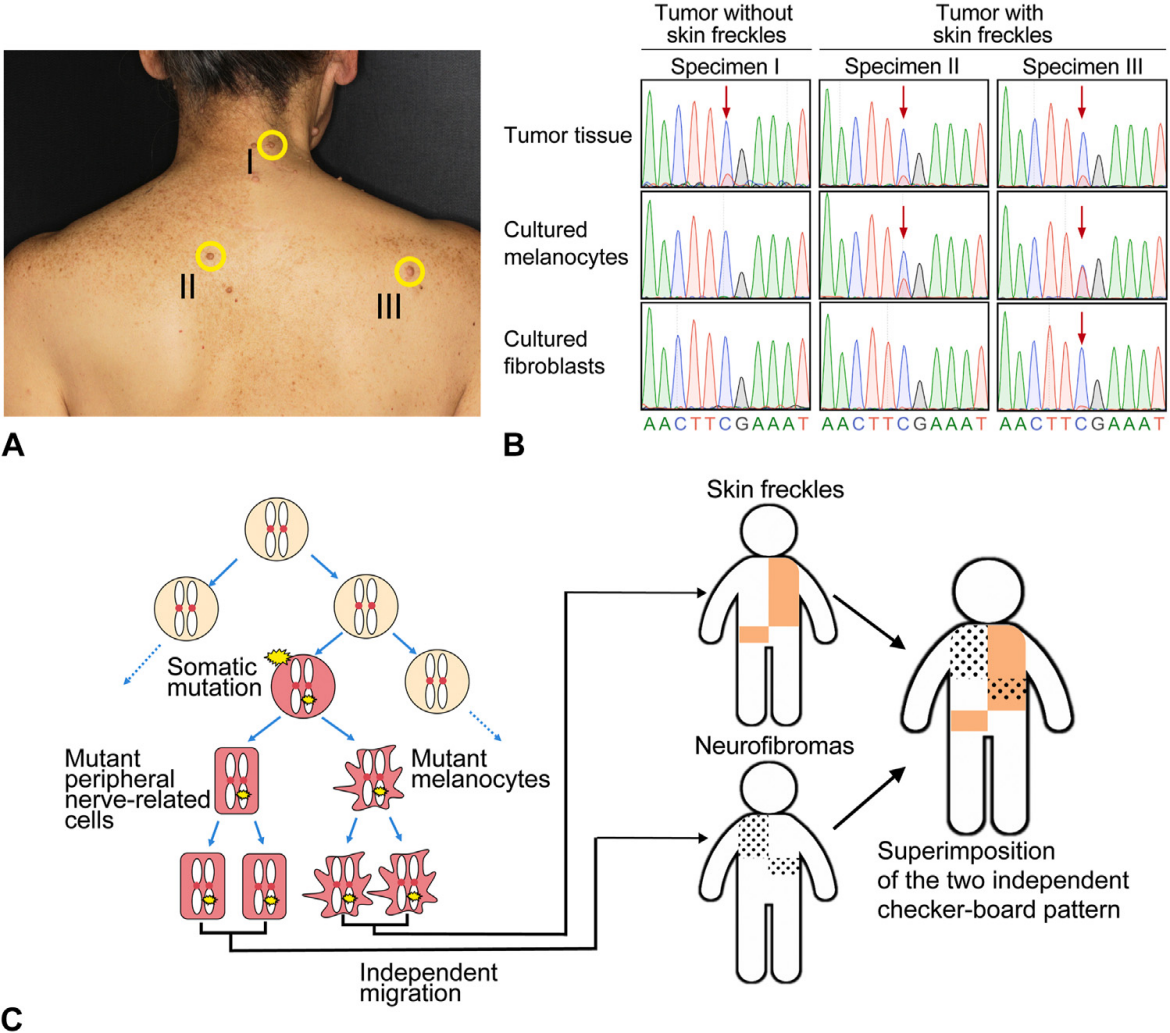
Genetic analyses**. A,** Biopsies of tumors from skin without ephelides (I) and from skin with ephelides (II and III). **B,** A Sanger sequencing chromatogram showing the c.1318C>T mutation of *NF1* (*red arrows*) in genomic DNA isolated from the indicated samples. **C,** A schematic illustrating how the 2 independent checkerboard patterns of the ephelides and neurofibromas became superimposed. Mutant melanocytes and PNR cells derived from an *NF1-*mutated pluripotent cell migrated independently and then formed neurofibromas (*dotted area*) and ephelides (*brown area*), respectively. The clinical phenotype is a superimposition of both lesions.

Both neurofibromas and ephelides were evident on the right upper part of her back, but the right part of her neck featured only neurofibromas and her left neck only ephelides (Fig 1, *D* and *E*), indicating that the checkerboard patterns of the ephelides and neurofibromas were distinct but partly superimposed. To investigate causative genetic changes, we excised 2 skin tumors from an area featuring ephelides and 1 tumor from an area lacking ephelides (Fig 2, *A*). The epidermis and dermis were separated via dispase treatment.^7^ The intradermal tumors were physically isolated. Primary cultures of melanocytes and fibroblasts were established from the isolated epidermis and dermis, respectively, as described previously.^7^ Genomic DNA was purified from the isolated tumors and the primary cultures. Using Sanger sequencing, a mosaic somatic *NF1* c.1318C>T mutation was detected in all 3 tumors. The mutation was present (at a high mosaic ratio) in primary cultures of melanocytes from ephelis-affected but not from ephelis-free skin (Fig 2, *B*). The mutation was barely present in primary cultures of fibroblasts from both ephelis-free and -affected skin (Fig 2, *B*). Thus, *NF1-*mutated melanocytes were present in ephelis-affected but not ephelis-free skin, independently of the distribution of neurofibromas.

## Discussion

SNF is caused by a prenatal somatic mutation in *NF1*. Café-au-lait spots and ephelides originate from melanocytes with the *NF1* mutation, while neurofibromas originate from mutated, PNR cells.^5^^,^^6^^,^^8^ As both melanocytes and PNR cells develop from ectodermal cells of the neural crest, an SNF-causing somatic mutation occurs in a pluripotent cell, which proliferates while maintaining pluripotency and then differentiates (within the neural crest) into the precursors of melanocytes and PNR cells. In mice, most melanoblasts develop from the neural crest and commence migration on embryonic day 10.5, whereas the Schwann cell precursors that cause neurofibromas differentiate from the neural crest on approximately embryonic day 12.^9^ Given this time course, the independent localization of ephelides and neurofibromas in our present case suggests that the mutant melanocytes and mutant cells that caused the neurofibromas migrated independently from the neural crest at different developmental time points, explaining the independent distributions of the ephelides and neurofibromas (Fig 2, *C*).

In our present case, the mosaic *NF1* mutation was also detected in peripheral blood leukocytes originating from mesoderm, suggesting that the disease-causing *NF1* mutation occurred in a pluripotent cell prior to the differentiation of ectoderm and mesoderm from the epiblast. Fibroblasts originate from mesoderm. The *NF1* mutation was undetectable or only barely detectable in fibroblasts cultured from biopsied specimens, suggesting that the ratio of *NF1*-mutated cells was low in fibroblasts of the biopsied skin. *NF1*-mutated fibroblasts are thought to be distributed independently of the mutated melanocytes and PNR cells. *NF1*-mutated fibroblasts are possibly clustered in some skin areas; however, since no skin symptoms have been established to be caused by *NF1*-mutated fibroblasts alone, it is challenging to locate such skin areas. The patient developed multiple GISTs, which are mesenchymal neoplasms, the incidence of which is increased in patients with NF1.^10^ Mesenchymal cells generally originate from mesoderm, suggesting that the GISTs developed from mesodermal cells that bore the somatic *NF1* mutation.

We present a case of SNF exhibiting superimposed but independent checkerboard patterns of ephelides and neurofibromas. Detailed analyses revealed that these patterns were attributable to the independent distributions of *NF1*-mutated melanocytes and PNR cells during development. The various combinations/distributions of clinical phenotypes include ephelides, neurofibromas, GISTs, and malignant peripheral nerve sheath tumors, reflecting when, and in which pluripotent cell, the causative somatic mutation occurred during development and the destinations of the daughter mutant cells. This case suggests to clinicians that genetic testing from cultured fibroblasts alone is at risk of false-negative results in the clinical diagnosis of SNF and that the risk of internal malignancies such as GISTs is not associated with the distribution of superficial skin symptoms, such as the presence of ephelides or neurofibromas on the abdominal skin, because the causative mutant cells migrate and are distributed independently in each organ. Genetic analyses of multiple cells/tissues from each germ layer enhance our understanding of the phenotypic diversity of SNF and enable evaluation of possible future complications.

## Conflicts of interest

None disclosed.
